# East Coast Fever Carrier Status and *Theileria parva* Breakthrough Strains in Recently ITM Vaccinated and Non-Vaccinated Cattle in Iganga District, Eastern Uganda

**DOI:** 10.3390/pathogens12020295

**Published:** 2023-02-10

**Authors:** Stephen Oligo, Ann Nanteza, Julius Nsubuga, Abubakar Musoba, Anne Kazibwe, George Willy Lubega

**Affiliations:** College of Veterinary Medicine, Animal Resources and Biosecurity (COVAB), Makerere University, Kampala P.O. Box 7062, Uganda

**Keywords:** *Theileria parva*, carrier, Muguga cocktail, genetic diversity, infection and treatment method, breakthrough

## Abstract

East Coast fever (ECF) is a tick-borne disease of cattle that hinders the development of the livestock industry in eastern, central and southern Africa. The ‘Muguga cocktail’ live vaccine, delivered by an infection and treatment method (ITM), remains the only immunisation strategy of controlling ECF. However, there are challenges of the live vaccine inducing ECF carrier status in immunised animals and the possibility of lack of protection from parasite strains that are antigenically different from the vaccine strains. In Uganda, there are insufficient data regarding the ECF carrier status and *T. parva* genetic diversity in vaccinated and associated non-vaccinated cattle to assess the effectiveness of ITM vaccination. Blood was collected from recently ECF vaccinated (98) and non-vaccinated (73) cattle from Iganga district in Eastern Uganda at 120 days post-vaccination. The p104 gene nested PCR was used to screen for *T. parva* DNA, 11 minisatellite and 3 microsatellite markers (SSR) were used for genotyping. Two minisatellite markers (MS7 and MS19) were used to determine whether ECF carrier status was due to the *T. parva* vaccine or local strains. The prevalence of *T. parva* based on p104 nPCR was 61.2% (60/98) (RR 2.234, 95% CI 1.49–3.35, *p*-value < 0.001) among recently vaccinated cattle and 27.4% (20/73) (RR 1.00) among associated non-vaccinated cattle. The Muguga cocktail vaccine strains were responsible for carrier status in 10 (58.8%) by MS7 and 11 (64.7%) by MS19 in vaccinated cattle. Genotypes of *T. parva* with different-sized alleles to the vaccine strains that could be potential ‘breakthroughs’ were detected in 2 (11.8%)) and 4 (23.5%) isolates from vaccinated cattle based on MS7 and MS19 minisatellite markers, respectively. Using 14 SSR markers, *T. parva* diversity was higher in vaccinated (Na = 2.214, Ne = 1.978, He = 0.465) than associated non-vaccinated (Na = 1.071, Ne = 1.048, He = 0.259) cattle. The principal component analysis (PCA) showed isolates from vaccinated cattle were closely related to those from non-vaccinated cattle. The analysis of molecular variance (AMOVA) revealed high genetic variation (96%) within *T. parva* isolates from vaccinated and non-vaccinated cattle but low variation (4%) between vaccinated and non-vaccinated cattle. This study reveals the role of ITM in inducing the carrier status and higher *T. parva* genetic diversity in vaccinated cattle. The low genetic variation between *T. parva* isolates in both vaccinated and non-vaccinated cattle may be suggestive of the protective role of vaccine strains against genetically related local strains in the study area.

## 1. Introduction

East Coast fever (ECF) is a tick-borne disease of cattle which is caused by a hemoprotozoan parasite called *Theileria parva (T. parva*). The parasite is transmitted by a brown ear tick (*Rhipicephalus appendiculatus)* and it is highly prevalent in eastern, central and southern Africa. East Coast fever (ECF) ranks first among the important tick-borne diseases of cattle in the region and causes high mortality, especially in exotic and cross-bred cattle, as well as indigenous calves below 6 months of age. The annual losses from TBD in Tanzania were estimated to be USD 364 million, and 1.3 million was the estimated mortality in cattle where theileriosis accounted for 68% of losses [1].

In Uganda, ticks and tick-borne diseases (TTBDs) are wide spread and they are a major threat to cattle, causing substantial mortality and reduced production [2,3,4]. Previous studies conducted in Mbarara and Tororo districts indicated ECF prevalence of 19.8% and 5.3%, respectively, using conventional PCR [2,5]. East Coast fever is the leading cause of economic losses to farmers in terms of costs incurred through cattle vaccination, tick control by use of acaricides and anti-theilerial drugs, in addition to losses via cattle weight loss, cattle mortality and milk reduction. About UGX 400 million in Uganda are spent annually on controlling TTBDs [6].

One of the promising and sustainable methods of protecting cattle against ECF is vaccination by infection and treatment method (ITM), which involves inoculation of cattle with live *T. parva* sporozoites and simultaneous administration of an antibiotic called oxytetracycline to curtail the infection [7]. The most widely used vaccine cocktail stabilate is the ‘Muguga cocktail’, which is composed of *T. parva* Muguga, Serengeti-transformed and Kiambu 5 stocks [8]. However, the vaccine requires a liquid nitrogen cold chain for its storage and delivery to farmers and is a potential lethal product if not given with adequate antibiotic cover. The cocktail combination has been used in Uganda, Malawi, Tanzania, Kenya and South Sudan [9].

Widespread deployment of ITM, however, has been hindered by several concerns [10]. The vaccine induces a carrier state in immunised animals which is described as the ability of an infected and recovered host to infect ticks which are then able to transmit the parasite to susceptible hosts [11]. *Theileria parva* carrier status can lead to the introduction and eventual dominance of the novel parasite genotypes derived from the vaccine into the areas previously free of these genotypes. This can complicate the genetic structure of *T. parva* when vaccine strains undergo genetic recombination with the local strains in the vector tick. If this occurred, it would hinder the use of the current Muguga cocktail vaccine in the control of ECF [10]. A carrier state may be important in maintaining immunity [12]. Studies have shown that the Kiambu 5 component of the vaccine causes a long-term carrier state of more than 87 days post vaccination, whereas Muguga and Serengeti-transformed components cause short-term carrier status [10,12]. The other concern pertaining the ITM use is that there is also the possibility of ‘breakthroughs’ from parasite strains antigenically distinct from the vaccine strains. The ‘breakthrough’ has been defined as the persistence of *T. parva* strain(s) in the ITM vaccinated cattle, antigenically distinct from those in the vaccine strains [13]. Breakthrough strains cause vaccinated cattle to develop the clinical signs of ECF, yet the role of the vaccine is to protect the cattle from ECF. If this occurred, it would require the incorporation of additional stocks into the vaccine.

The identification of satellite sequences from the genome of *T. parva* enabled comprehensive studies to be completed on the genetic relationship among *T. parva* populations [14,15]. These satellite markers also enabled studies to be performed on *T. parva* population dynamics and sub-structuring of field isolates [16]. The micro- and minisatellite markers’ sequences comprise of multiple nucleotide repeats, which, because of varying numbers of repeats, give rise to alleles with distinct amplicon sizes. These markers were also used to elucidate the impact of immune selection on the parasite [17] and the risk associated with the ITM [10,13]. In Uganda, data regarding ECF carrier status and threat from ‘breakthroughs’ following ITM vaccination are limited. This study, therefore, aimed at demonstrating the establishment of carrier status and occurrence of ‘breakthroughs’ and associated genetic diversity of *T. parva* among the ITM vaccinated and associated non-vaccinated cattle in Iganga district, eastern Uganda. The study determined the prevalence of *T. parva* in recently vaccinated and associated non-vaccinated cattle, which demonstrated whether the ECF carrier status was caused by vaccine or local strains in the vaccinated cattle and the occurrence of *T. parva* ‘breakthroughs’ in the vaccinated cattle. The *T. parva* genetic diversity was assessed from the vaccinated and non-vaccinated cattle. Data generated from this study provide additional information on the establishment and persistence of carrier status and genetic diversity of *T. parva* in the vaccinated and non-vaccinated cattle in an ECF endemic area. This is important for the use of ITM in the integrated control of ECF in endemic areas.

## 2. Materials and Methods

### 2.1. Study Area

**Figure 1:**
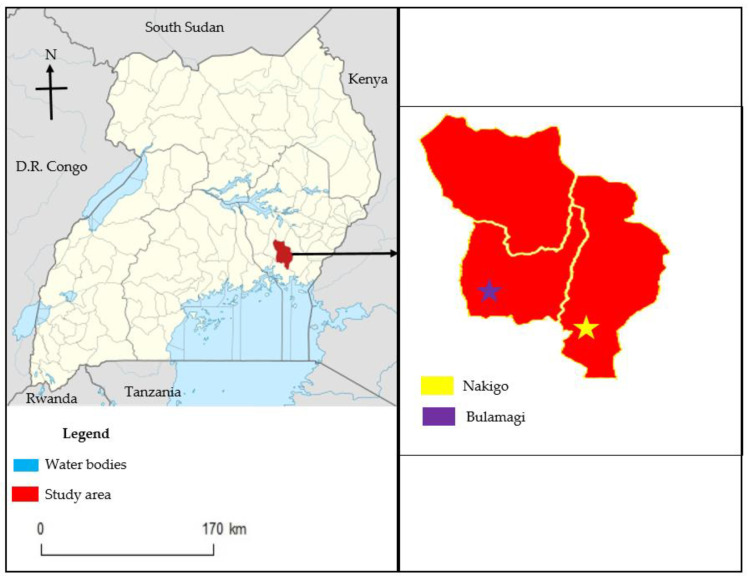
Map of Uganda showing Iganga district and location of study sub-counties. Map of Uganda was adopted from Uganda bureau of statistics and modified.

The study was conducted in Bulamagi and Nakigo sub-counties in Iganga district in the eastern region of Uganda (Figure 1). The coordinates for Iganga district are: Latitude: 0°36′33.01″ N, Longitude: 33°28′7.00″ E. Districts along its boarders are Bugiri to the east, Namutumba to the northeast, Kaliro to the north, Kamuli to the northwest, Jinja to the west, and to the south lies Mayuge. The district has an average annual temperature of 22.3 °C and an average annual rainfall of 1313 mm, which are optimal for the survival of the livestock and disease vectors such as ticks. There are two relatively drier seasons (December to March and June to July) [18]. There is sufficient rainfall throughout the year to sustain man and animals except in very rare circumstances that sometimes lead to drought.

### 2.2. Study Design

This was a cross-sectional study that involved sampling of cattle based on the ECF-ITM vaccination status, whereby both recently vaccinated (120 days post-vaccination using Muguga cocktail vaccine) and non-vaccinated cattle from the same farms. Vaccination status was confirmed from farmer records and special ear tags were put on the ECF vaccinated cattle. Fifteen cattle farms were selected from two sub-counties (Nakigo and Bulamagi) in Iganga district. Blood (5 mL) was drawn from the jugular vein into the EDTA vacutainer tube and placed in a cool box for short-term storage and, thereafter, transported on ice to the Molecular Biology Laboratory (MOBILA), College of Veterinary Medicine, Animal Resources and Biosecurity (COVAB) for laboratory analysis.

Genomic DNA was extracted from cattle whole blood using the purelink^TM^ genomic DNA mini kit (Invitrogen, Waltham, MA, USA). The DNA concentration and purity were determined using UV spectrophotometry (nanodrop) at 260–280 nm. The DNA was screened for presence of *T. parva* parasites using primers designed from p104 gene by nested PCR [19]. Positive p104 nPCR *T. parva* isolates were genotyped using fourteen satellite markers (SSRs): 11 mini- (MS) and 3 microsatellite (ms) markers; (MS7, MS19, MS3, MS16, MS8, MS21, MS25, MS27, MS33, MS34, MS40, ms2, ms5, and ms7) [15]. The data generated were analysed to determine the prevalence, genetic diversity and persistence of carrier status of *T. parva* in recently vaccinated and associated non-vaccinated cattle in Iganga district. The MS19 and MS7 satellite markers were used to assess whether the carrier status was due to the vaccine or local parasite strains.

### 2.3. Recruitment and Vaccination of Animals

The animals were recruited into the study after a written informed consent by the cattle farmers. The inclusion criteria were based on animals ≥ 8 months old without fever (rectal temperature < 39.5 °C) and non-pregnant for the female animals. Cattle were ear-tagged and weighed. The immunisation procedure was carried out as described previously [8,20]. The immunised animals were injected with 1 mL of 100× dilution of MCL01 stabilate. The vaccine was inoculated subcutaneously in front of the right parotid lymph node and the animals were treated simultaneously with 30% long acting Oxytetracycline (Tetroxy LA, Bimeda, Dublin, Ireland) at a dose rate of 30 mg/kg body weight by deep intramuscular injection. The vaccine used was the trivalent formula known as Muguga Cocktail composed of Muguga, Kiambu-5 and Serengeti-transformed stocks [21]. The vaccine was purchased from SCOPEVET, (an authorised ECF vaccine importer/supplier in Uganda). The animals were monitored daily for 28 days post ITM vaccination for detection of clinical symptoms by the farmers and the Resident Veterinary Officers.

### 2.4. Detection of p104 Gene and Genotyping of Theileria parva Isolates Using Mini- and Microsatellite Markers by Nested PCR

Genomic DNA was extracted from 160 µL of EDTA vacutainer collected cattle blood using the purelink^TM^ genomic DNA mini kit (Invtrogen, USA) according to manufacturer’s instructions. The extracted DNA was stored at −20 °C until needed for *T. parva* screening and genotyping using nested PCR with p104 and SSR primers (Eurofins Genomics at GmbH, Viehmarktgassen 1B/Büro, AT-1030 Vienna, Austria). The nPCR for an invariable region of the p104 gene [19] was used to detect *T. parva* DNA in the extracted DNA test samples along with positive and negative controls. A negative control was a PCR reaction master mix with distilled water as DNA template. *Theileria parva* Muguga stock genomic DNA from International Livestock Research Institute (ILRI) was used as positive control.

**Figure 2:**
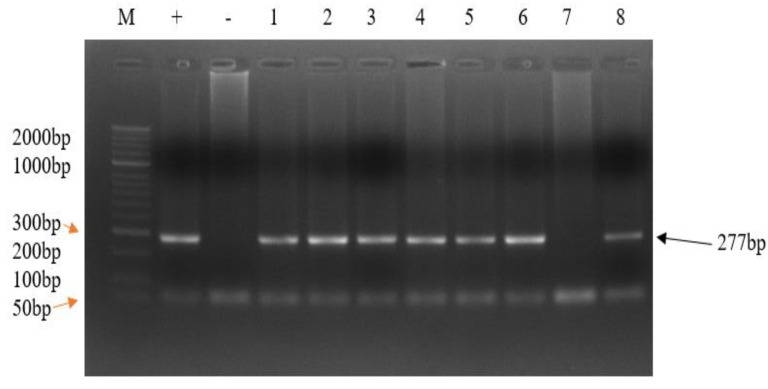
A representative 2% agarose gel showing *T. parva* p104 gene nPCR amplicons. Lane M: 50bp DNA ladder (N32361, Biolabs), Lane +: positive control (*T. parva* Muguga stock DNA), lane −: negative control (distilled water), lanes 1–6 and 8: *T. parva* positive samples Lane 7: *T. parva* negative sample.

For detection of p104 gene for *Theileria parva*, primary PCR amplification of the p104 gene generated a 496bp fragment using forward and reverse primers IL3231 (5’-ATTTAAGGAACCTGACGTGACTGC-3’) and IL755 (5’-TAAGATGCCGACTATTAATGACACC-3’), respectively, as described by Skilton et al. [22]. Briefly, 2× Taq kappa with dye was used with the final volume of 10 µL; containing 5.0 µL 2X Taq kappa with dye, 0.25 µL of each forward and reverse primers, 2.5 µL of PCR grade nuclease-free water and 2.0 µL of the DNA template. The PCR conditions for amplifying the p104 gene in the primary PCR were as follows; initial denaturation at 94 °C for 5 min, followed by 40 cycles of denaturation at 94 °C for 1 min, primer annealing at 58 °C for 1 min, extension at 72 °C for 1 min. Final extension at 72 °C for 9 min and hold at 4 °C. The secondary PCR amplified a 277bp internal fragment located between bases 2784 and 3061 of the p104 gene using forward and reverse primers 5’-GGCCAAGGTCTCCTTCAGATTACG-3’ and 5’-GTGGGTGTGTTTCCTCGTCATCTGC-3’, respectively, as described by Odongo et al. [19]. The primary PCR product (1.0 µL) was used as a DNA template in a 10 µL reaction volume for the secondary PCR as above. The PCR conditions for amplifying the p104 gene in the secondary PCR were as described above except annealing temperature of 60 °C for 1 min. The secondary PCR products (5 µL) were checked on 2% agarose gel in Tris-acetate-EDTA (TAE) buffer at 125 V and 300 A for 40 min. Band size was determined using a 50 bp DNA ladder (N32361, Biolabs). The DNA bands were visualised under UV light, photographed and documented (Figure 2).

For genotyping *T. parva,* positive p104 PCR samples and the Muguga cocktail vaccine DNA were used. The 3 micro- (ms2, ms5, ms7) and 11 minisatellite markers (MS3, MS5, MS7, MS16, MS19, MS2, MS25, MS27, MS33, MS34, MS40) [15] were used. Positive (*T. parva* Muguga stock DNA) and negative (distilled water) controls were run together with test samples. The MS7 and MS19 satellite markers were used to assess whether the *T. parva* carrier status was due to the vaccine or local strains [10,15,23].

**Figure 3:**
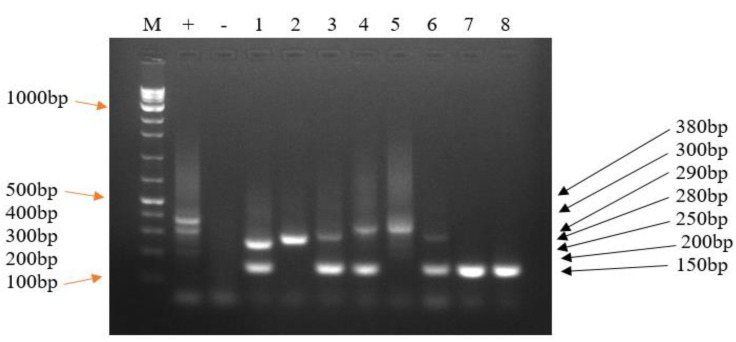
A representative 2% agarose gel showing *T. parva* nPCR amplicons using MS7 marker. Lane M: 100bp ladder (Biolabs), Lane +: positive control (Muguga cocktail vaccine isolate DNA which amplified three alleles of 200 bp, 300 bp, 380 bp), lane −: negative control (distilled water), Lanes 1–8: field sample isolates. Lanes 1, 2, 3, 5 and 6 were from vaccinated cattle whereas Lanes 4, 7 and 8 were from non-vaccinated cattle isolates. The DNA from field isolates (1–8) amplified five alleles, 150 bp (Lanes 1, 3, 4, 6, 7 and 8), 250 bp (Lane 1), 280 bp (Lane 2), 290 bp (Lane 3 and 6), and 300 bp (Lanes 4 and 5).

The reaction mixture volumes used in primary PCR were as described above for detection of p104. The PCR conditions were set as follows; initial denaturation at 94 °C for 5 min, followed by 40 cycles of denaturation at 94 °C for 1 min, annealing at 55 °C for 1 min, extension at 65 °C for 1 min, final extension at 65 °C for 9 min and hold at 4 °C. The reaction mixture volumes used in secondary PCR were as described above in the nested p104 amplification. The secondary PCR conditions were set as described above in primary genotyping except using annealing temperature of 58 °C for 1 min. The secondary PCR products (5 µL) were analysed as described above for p104 detection (Figure 3).

### 2.5. Data Analysis and Interpretation

Data generated from screening for *T. parva* DNA using p104 nPCR and genotyping using satellite markers (SSR) were entered and cleaned using Microsoft Excel. Descriptive statistics were computed at 95% confidence interval. Data from screening were analysed using Chi-square test to determine the association between outcome variable (*T. parva* positivity) and categorical variable (vaccination status) at statistical significance *p* < 0.05.

Data from genotyping were analysed using GenALEX software version 5 [24], which was used to calculate genetic diversity parameters. This included determining the mean number of alleles (Na), number of effective alleles (Ne) and expected heterozygosity (He). These parameters were used to determine parasite diversity (overall and within the parasite populations from vaccinated and non-vaccinated cattle). Principal Component Analysis (PCA) was used to determine the genetic relationships among *T. parva* isolates from Muguga cocktail vaccine, vaccinated and non-vaccinated cattle. Analysis of molecular variance (AMOVA) was also used to determine *T. parva* diversity by estimating the percentage variation within the individual population and between the two populations (vaccinated and non-vaccinated).

The detection of *T. parva* DNA in the vaccinated or non-vaccinated cattle blood was referred to as ‘carrier’ status since clinical ECF was not observed in the study animals. The appearance of parasite strain/genotype in the vaccinated cattle that was not similar to vaccine strains was considered as ‘breakthrough’.

## 3. Results

### 3.1. Farm and Farmer Characteristics

The cattle sampled (n = 171) were from fifteen farms from Iganga district in Eastern Uganda and they were kept under semi-intensive or zero grazing farming systems. The majority of the cattle sampled were 160 (93.6%) adults, 154 (90.1%) Friesian cross breed and 141 (82.5%) females (Table 1).

### 3.2. Prevalence of Theileria parva by p104 nPCR from ITM Vaccinated and Non-Vaccinated Cattle

The isolates which amplified with band size of 277bp were positive for *T. parva* parasite (Figure 2). The prevalence among vaccinated cattle was 61.2% (60/98) and non-vaccinated cattle was 27.4% (20/73) (Table 2). The overall prevalence of *T. parva* was 46.8% (80/171).

### 3.3. Determination of the Source of Theileria parva Genotypes in ECF Vaccinated Cattle

In order to determine whether *T. parva* carrier status in recently vaccinated cattle was due to the vaccine or local strains, 80 positive p104 nPCR isolates were genotyped; however, only 32.5% (26/80) *T. parva* field isolates gave amplicons with both the MS7 and MS19. Using MS7 minisatellite marker, 58.8% (10/17) of *T. parva* DNA from vaccinated cattle amplified alleles of the same size as that amplified by the Muguga vaccine DNA (300 bp). The extracted DNA samples from non-vaccinated cattle (44.4% (4/9)) amplified alleles of the same size as that amplified by the Muguga cocktail vaccine (300 bp) and 55.6% (5/9) of the non-vaccinated cattle showed an allele of 150bp, which also appeared in 29.4% (5/17) of DNA from vaccinated cattle. The allele band size (150 bp) was predominant in local strains and absent in the cocktail vaccine. Some isolates from vaccinated cattle 11.8% (2/17) amplified an allele of 280 bp, which was not amplified in the majority of the isolates from non-vaccinated cattle and also not detected in the Muguga cocktail vaccine strains. The 280 bp allele, when detected in vaccinated cattle, was assumed to be a breakthrough strain. Two alleles (200 bp and 380 bp) were amplified from the cocktail vaccine DNA but were neither amplified from isolates from vaccinated nor non-vaccinated cattle. Many isolates from vaccinated cattle (47.1% (8/17)) had more than one allele, indicative of multiple infections by many parasite strains. A total of 33.3% (3/9) of isolates from non-vaccinated cattle also had two alleles each (Figure 3).

**Figure 4:**
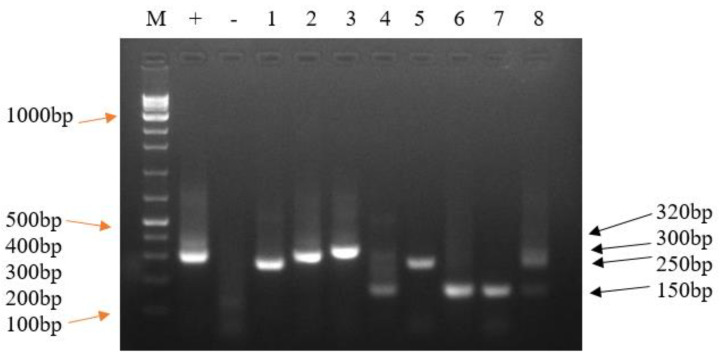
A representative 2% agarose gel showing *T. parva* nPCR amplicons using MS19 marker. Lane M: 100 bp ladder (New England Biolabs, Ipswich, MA, USA), Lane +: positive control (Muguga cocktail vaccine isolate DNA which amplified one allele (300 bp), lane −: negative control (distilled water), Lanes 1–8: field sample isolates. Lanes 1, 2, 3, 5 and 6 were from vaccinated cattle whereas Lanes 4, 7 and 8 were from non-vaccinated cattle isolates. The DNA from field isolates when genotyped with minisatellite marker (MS19) amplified four alleles of sizes; 150 bp (Lanes 4, 6, 7 and 8), 250 bp (Lane 1), 300 bp (Lanes 2, 4, 5 and 8) and 320 bp (Lane 3).

Genotyping with MS19 minisatellite marker showed that 64.7% (11/17) of *T. parva* isolates from Muguga cocktail-vaccinated cattle amplified alleles of the same size as those amplified by the Muguga cocktail vaccine (300 bp) and 33.3% (3/9) of isolates from non-vaccinated cattle also amplified similar allele size. The non-vaccinated cattle parasite isolates (66.7% (6/9)) amplified a predominant allele of 150 bp, which was also amplified by 11.8% (2/17) of the vaccinated cattle parasite isolates. Parasite isolates from vaccinated cattle (23.5% (4/17)) amplified an allele of either 250 bp (2/4) or 320 bp (2/4), which were neither amplified from the non-vaccinated cattle nor the vaccine DNA, which could present a possible ‘breakthrough’ strain (Figure 4).

### 3.4. Determination of Theileria parva Genetic Diversity in Vaccinated and Non-Vaccinated Cattle Using Mini- and Microsatellite Markers

Eighty positive p104 isolates were genotyped using 14 SSR (11 minisatellite and 3 microsatellite) markers spanning the four *T. parva* chromosomes. Only 32.5% (26/80) of isolates showed signals with all 14 primer sets considered for analysis. The total number of alleles in the parasite isolates from vaccinated cattle ranged from three to five; three (MS27, MS16, MS27, MS40, MS33, MS25, MS3, ms2, ms7, ms5), four (MS19, MS34, MS8), and five (MS7), whereas the number in non-vaccinated cattle parasite isolates ranged from two to four; two (MS16, MS21, MS40, MS25, ms2, ms5), three (MS19, MS27, MS34, MS33, MS8, MS3, ms7), and four (MS7). The Muguga cocktail vaccine sample amplified with either one (MS19, MS34, MS40, MS33, MS8, MS3, MS25, ms7, ms5), two (MS27, MS16, MS21, ms2), or three (MS7) alleles. All the genetic diversity parameters; the mean number of the different alleles (Na), mean number of effective alleles (Ne) and expected heterozygosity (He) were higher in Muguga cocktail-vaccinated than non-vaccinated cattle (Table 3).

### 3.5. Determination of the Genetic Relationship between Theileria parva Isolates from Vaccinated and Non-Vaccinated Cattle

**Figure 5:**
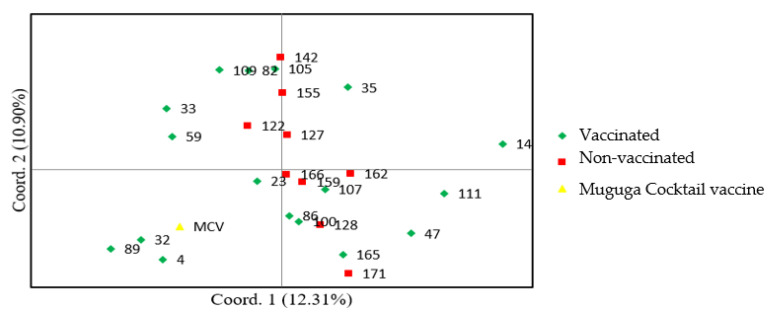
Principal component analysis (PCA) applied to VNTR alleles within *T. parva* populations in ECF vaccinated and non-vaccinated cattle from Iganga district, Eastern Uganda. The analysis was based on genotype data from 14 VNTR (11 mini- and 3 micro-satellite) loci. Each square represents an individual parasite allele. Each colour represents a study group as depicted from the key.

In the principal component analysis (PCA) plot, there was no distinct clustering as the parasite alleles scattered throughout the plot. However, most of the parasite alleles from vaccinated and non-vaccinated cattle appeared in the two upper and lower right quadrants. The lower left quadrant contains alleles from vaccinated cattle and Muguga cocktail vaccine (Figure 5).

### 3.6. Determination of the Genetic Variation of Theileria parva Parasites from Vaccinated and Non-Vaccinated Cattle Populations

Analysis of molecular variance (AMOVA) calculated from the two parasite populations revealed a large percentage of genetic variation (96%) within individual populations with only 4% being explained by differences among populations (Table 4).

## 4. Discussion

This study investigated the carrier status and genetic diversity of *T. parva* in recently vaccinated cattle of 120 days post vaccination. There was an overall *T. parva* prevalence of 46.8% in cattle sampled from two sub-counties in Iganga district. The prevalence was higher in recently Muguga cocktail-vaccinated cattle (61.2%) than non-vaccinated cattle (27.4%). Previous studies conducted in Tanzania also reported similar results where prevalence of *T. parva* was higher in vaccinated cattle than non-vaccinated cattle [25,26]. More than half of the recently vaccinated cattle became carriers of *T. parva*, justifying the importance of ITM since the majority of the vaccinated cattle become carriers and remain immune to ECF [27]. The persistence of carrier state for up to 120 days post vaccination was probably a combined effect of vaccination and the continuous tick challenge to cattle under natural field conditions exposure. This is confirmed by the fact that tick exposure plays a major role in form of an incremental booster effect on the ITM-induced immunity [25].

The *T. parva* carrier status was not detected in all the recently vaccinated cattle. Failure to detect the carrier state in all vaccinated cattle, however, does not necessarily mean vaccine failure or lack of immunity in an animal, because some cattle after vaccination may clear the parasite and remain immune in absence of a carrier state, a condition known as sterile immunity [10,28]. It is also possible that the parasite may be present in other tissues such as lymph nodes, other than blood [28]. However, vaccination failure can also occur whereby a high dose of long-acting Oxytetracycline is administered during the ITM vaccination and may clear all the infection. Alternatively, the carrier status may wane when not boosted by infected ticks under natural tick challenge. None of the vaccinated cattle showed clinical signs of ECF disease, implying that the vaccine protected them. In the case of the non-vaccinated cattle, the natural infection may have induced a carrier status that elicits innate or natural immunity. The induced carrier status by natural infection is boosted and maintained by feeding infected ticks to the animals, thus providing natural immune protection. In addition, the improved farm management could have influenced the outcome of the lack of clinical symptoms, since farmers adhered to the appropriate use of acaricides to control the ticks during the study period, which may have kept the parasitaemia low. However, ECF is known to be a prevalent disease in the study area. Screening of non-vaccinated cattle using a nested p104 PCR revealed *T. parva* prevalence of 27.4% (20/73), which was much lower than vaccinated cattle. The non-vaccinated cattle could be carriers of the local strains of *T. parva* circulating in the field or they could be carriers of vaccine strains since it is possible for the local tick population to become infected with the vaccine parasite strains from vaccinated animals with resultant transmission to the non-vaccinated cattle which share grazing grounds [10]. There was, however, a significant difference in proportion of carriers between vaccinated and non-vaccinated cattle whereby prevalence of *T. parva* was higher in vaccinated cattle than non-vaccinated cattle, which confirms the impact of live vaccination in inducting a carrier state [26].

In order to answer the question of which strains of *T. parva* persisted in the cattle population after immunisation with Muguga cocktail vaccine, polymorphisms in the minisatellite markers MS7 and MS19 were studied. The MS7 and MS19 SSR markers were chosen because of their high sensitivity and their ability to differentiate between genotypes of *T. parva* from vaccine and field strains [16]. Some of the alleles which occurred in the Muguga cocktail vaccine DNA were detected in both vaccinated and non-vaccinated cattle. The appearance of the vaccine alleles in non-vaccinated cattle implies that such alleles may have been transmitted by ticks from vaccinated to non-vaccinated cattle or some of the local strains of *T. parva* circulating in the cattle population are similar to the vaccine strains. There were some alleles with band sizes of 200 bp and 380 bp which occurred in the Muguga vaccine with MS7 primers but were not detected in isolates from vaccinated cattle. These alleles appearing in the vaccine but were absent in the vaccinated cattle could be *T. parva* Serengeti-transformed stock and Muguga stocks since the Muguga vaccine is a cocktail of three main stocks; Muguga, Kiambu 5 and Serengeti-transformed [15]. The Muguga and Serengeti-transformed stocks induce a short-term carrier state of up to 87 days post vaccination, whereas Kiambu 5 induces a long-term carrier state [10,13]. The predominant alleles that were occurring in the majority of the non-vaccinated and in some of the vaccinated cattle isolates that are different from the Mugaga vaccine strains could possibly be local strains of *T. parva* circulating in the field. A study conducted by Oura et al. [10] also found that the vaccinated cattle can become infected with some local strains of *T. parva* in the field but these do not result in overt disease. However, there were alleles occurring in some isolates from vaccinated cattle that were neither observed in the vaccine nor from non-vaccinated cattle isolates, which could be possible ‘breakthrough’ strains [13].

Genotyping of *T. parva* isolates gave 3–5 alleles from vaccinated and 2–4 alleles from non-vaccinated cattle. The presence of numerous parasite allelic components reflects great antigenic heterogeneity and hence broadens the protection induced by vaccination. Individual parasite DNA isolates, however, carried either only one or two alleles each. This reflects a phenomenon that only a limited subset of *T. parva* genotypes can be transmitted by ticks to cattle, although *T. parva* genotypes circulating in the field are highly diverse [29]. The mean number of different alleles, mean number of effective alleles and expected heterozygosity were used to compare *T. parva* diversity among parasite populations. All these parameters were high in vaccinated (mean number of different alleles, 2.214; mean number of effective alleles, 1.978; expected heterozygosity, 0.465) than non-vaccinated cattle (mean number of different alleles, 1.071; mean number of effective alleles, 1.048; expected heterozygosity, 0.259). This implies that *T. parva* parasite diversity in recently vaccinated cattle was higher than the diversity in non-vaccinated cattle. This confirms the positive role of ITM of vaccination in increasing the diversity of *T. parva* parasites, which may be enhanced through influence of sexual recombination and continuous tick challenge, which may lead to wider immune protection [30].

The principal component analysis (PCA) depicted close genetic relatedness of *T. parva* parasite isolates from recently vaccinated and non-vaccinated cattle since they always scattered together. This shows that there was sharing of strains between vaccinated and non-vaccinated cattle. This has been confirmed as a possible scenario in the field where ticks aid in transferring *T. parva* parasites between cattle that share common grazing ground [10,26]. However, isolates from vaccinated cattle were more closely related to the strains from Muguga cocktail vaccine, as shown by the clustering in the lower left quadrat (Figure 5). The low genetic variation of 4% among the two populations also confirms that the two populations shared similar genetic composition. The parasite isolates scattered in all the four quadrants, implying that the *T. parva* parasites were genetically diverse in the field, which could be induced by genetic recombination between field parasites and vaccine parasites [30]. The principal component analysis plot findings were consistent with the high level of variation existing within individual parasite isolates in a population (96%), implying that there was high genetic diversity in each individual population. This is similar to findings by Magulu et al. [26].

During genotyping of field isolates with minisatellite markers MS7 and MS19 to determine whether *T. parva* carrier status was due to vaccine strains or due to field strains, a Muguga cocktail vaccine DNA (Serial no. ECF MCL 0202 CTT BD DEC 14) was used as reference control. However, it was difficult to point out exactly which strain of *T. parva* was causing carrier status among the 58.8% (MS7) and 64.7% (MS19) of the vaccinated cattle isolates which were carriers of vaccine strain. This is because a Muguga cocktail vaccine is composed of three stocks of *T. parva*; Muguga stock, Kiambu 5 stock and Serengeti-transformed stock. However, studies have shown that only Kiambu 5 stock alleles can be detected in vaccinated cattle isolates by PCR after 87 days: therefore, its alleles were the only ones expected. For clarity, it may have been best to use individual vaccine cell line stock components as reference controls. However, this was impended by limited finances to purchase the individual vaccine cell lines, as the Muguga cocktail vaccine used was easy to access since the project was involved in the vaccination exercise of cattle in Iganga district. In addition, there is a need to isolate the antigenic region from non-vaccine *T. parva* strains detected in vaccinated and non-vaccinated cattle and sequence to determine their genetic make-up and compare with the known strains of *T. parva* to ascertain the possibility of ‘breakthrough’ strains. Additionally, other tests such as serology (antibody test) should be performed to ascertain whether the strains causing carrier status among vaccinated cattle could be from previous infection, thus carriers already probably existed if the cattle were previously vaccinated, especially for cattle farmers that had not documented properly. 

## 5. Conclusions

The study revealed that the prevalence of *T. parva* was high in both vaccinated (61.2%) and non-vaccinated (27.4%) cattle at 120 days post vaccination. This was brought about by the carrier status of *T. parva* following Infection and treatment method (ITM) of vaccination using Muguga cocktail vaccine. The *T. parva* carrier status provided immunity to a high number of vaccinated and non-vaccinated cattle since no clinical symptoms of disease were observed. The *T. parva* Muguga cocktail vaccine strains were responsible for carrier status in the majority of the recently vaccinated cattle; 58.8% (MS7) and 64.7% (MS19). The non-vaccine strains causing carrier status in vaccinated cattle could be field strains or breakthrough strains. In addition, there were peculiar strains that appeared in 11.8% (MS7) and 23.5% (MS19) of vaccinated cattle, which could likely be breakthrough strains. The high genetic diversity of *T. parva* was due to continuous natural tick exposure in the field, leading to parasite strain recombination generating more genotypes, which brings about the high parasite allelic diversity in vaccinated and non-vaccinated cattle that share common grazing ground.

## Figures and Tables

**Table 1:** Demographic characteristics and ECF-ITM vaccination status of study cattle.

Variable	Frequency (%)	95% CI
**Vaccination status**		
Non vaccinated Vaccinated	73 (42.7%) 98 (57.3%)	35.42–50.28 49.71–64.57
**Age (months)**		
Adults (>6 months) Calves (3–6 months)	160 (93.6%) 11 (6.4%)	88.69–96.42 3.57–11.30
**Breed**		
Friesian cross Zebu	154 (90.1%) 17 (9.9%)	84.52–93.75 6.24–15.47
**Sex**		
Female Male	141 (82.5%) 30 (17.5%)	75.94–87.49 12.50–24.05

Key: Friesian cross = Friesian × Zebu or Ankole cattle breed.

**Table 2:** Association of ITM vaccination and *T. parva* infection status in cattle.

Variable	Univariate Analysis		Bivariate Analysis			Multivariate Analysis		
	*T. parva* PCR Results							
Vaccination Status	Negative = 91	Positive = 80	cRR	95% CI	*p*-Value	aRR	95% CI	*p*-Value
Non-Vaccinated	53(58.2%)	20(25%)	1.00	-	-	1.00	-	-
Vaccinated	38(41.8%)	60(75%)	2.234	1.49–3.35	<0.001	1.84	1.22–2.79	0.004
**Age (months)**								
Adults (>6 months)	81(89%)	79(98.8%)	1.00	-	-	1.00	-	-
Calves (3–6 months)	10(10%)	1(1.3%)	0.184	0.028–1.200	0.077	0.315	0.052–1.88	0.206
**Sex**								
Female	68(74.73)	73(91.25)	1.00	-	-	1.00	-	-
Male	23(25.27)	7(8.75)	0.450	0.231–0.879	0.019	0.744	0.398–1.392	0.35
**Breed**								
Friesian cross	81(89.01)	73(91.25)	1.00			1.00	-	-
Zebu	10(10.99)	7(8.75)	0.868	0.480–1.570	0.641	0.850	0.479–1.507	0.579

Key: Friesian cross = Friesian × Zebu or Ankole cattle breed.

**Table 3:** Allelic mean values across *T. parva* parasite populations from vaccinated and non-vaccinated cattle.

Mean	Vaccinated	Non-Vaccinated
Na	2.214	1.071
Na Freq. ≥ 5%	2.214	1.071
Ne	1.978	1.048
I	0.748	0.371
No. Private Alleles	1.286	0.143
No. Comm Alleles (≤25%)	0.000	0.000
No. Comm Alleles (≤50%)	0.000	0.000
He	0.465	0.259

Na = Number of Different Alleles, Ne = Number of Effective Alleles, No. Private Alleles = Number of Alleles Unique to a single Population, He = Expected Heterozygosity.

**Table 4:** Genetic variation of *T. parva* parasites from vaccinated and non-vaccinated cattle populations.

Source	Df ^a^	SS ^b^	MS ^c^	Est. Var. ^d^	% ^e^
Among Populations	1	8.586	8.586	0.181	4%
Within Populations	50	216.183	4.324	4.324	96%
Total	51	224.769		4.505	100%

^a^ Degree of freedom, ^b^ Sum of squares, ^c^ Mean of squares, ^d^ Estimated variance, ^e^ Percentage variation.

## Data Availability

The datasets used to analyse during current study are available from the corresponding author on request.
